# 2-O-Methylhonokiol Suppresses 3T3-L1 Adipogenesis Through Metabolic Stress Signaling and Early Cell Cycle Restriction

**DOI:** 10.3390/ijms27146529

**Published:** 2026-07-22

**Authors:** Minghao Fu, Manish Kumar Singh, Gyuhui Kim, Kyung-Sik Yoon, Sung Soo Kim, Joohun Ha, Insug Kang, Wonchae Choe

**Affiliations:** 1 Department of Biochemistry and Molecular Biology, School of Medicine, Kyung Hee University, Seoul 02447, Republic of Korea; andrew1179609214@gmail.com (M.F.); sky9999@khu.ac.kr (K.-S.Y.); sgskim@khu.ac.kr (S.S.K.); hajh@khu.ac.kr (J.H.); iskang@khu.ac.kr (I.K.); 2 Department of Biomedical Science, Graduate School, Kyung Hee University, Seoul 02447, Republic of Korea; 3 Department of Precision Medicine, Graduate School, Kyung Hee University, Seoul 02447, Republic of Korea; eieiclo@khu.ac.kr

**Keywords:** 2-O-methylhonokiol, honokiol derivative, 3T3-L1 adipocytes, adipogenesis, lipid accumulation, AMPK, ER stress, IRE1α/XBP1s, kinase remodeling, mitotic clonal expansion

## Abstract

Excessive adipose tissue expansion contributes to metabolic dysfunction, highlighting the need to identify compounds that restrain adipocyte differentiation without causing nonspecific cytotoxicity. This study investigated whether 2-O-methylhonokiol suppresses adipogenesis in 3T3-L1 preadipocytes and examined its effects on adipogenic transcription, metabolic signaling, ER stress-related responses, kinase activity, and early cell cycle progression. 3T3-L1 cells were induced to differentiate with an MDI cocktail and treated with 25 or 50 µM 2-O-methylhonokiol during differentiation. Lipid accumulation was evaluated by Oil Red O staining, protein expression and phosphorylation were analyzed by immunoblotting, cell cycle distribution was assessed 24 h after MDI induction, and basal viability was measured using the CCK-8 assay. 2-O-methylhonokiol markedly reduced neutral lipid accumulation and suppressed key adipogenic regulators and maturation markers, including C/EBPβ, PPARγ, C/EBPα, FABP4, and FASN. At the signaling level, 2-O-methylhonokiol increased AMPKα phosphorylation without altering total AMPKα and reduced FASN expression, whereas mTOR phosphorylation was reduced at 50 µM. These findings suggest that 2-O-methylhonokiol is associated with metabolic and lipogenic signaling remodeling during adipocyte differentiation. 2-O-methylhonokiol also modified kinase-associated responses, increasing AKT and JNK phosphorylation while reducing the detectable phosphorylated ERK signal, with total AKT, ERK, and JNK remaining largely unchanged. ER stress-related signaling displayed a selective profile, characterized by increased IRE1α expression, XBP1s induction, elevated p-eIF2α, unchanged total eIF2α, and no compensatory increase in GRP78. During early adipogenesis, 2-O-methylhonokiol attenuated MDI-induced cell cycle redistribution by preserving a larger G0/G1 population and limiting progression toward later cell cycle phases, consistent with impaired mitotic clonal expansion. These effects occurred at concentrations that did not significantly reduce basal cell viability. Overall, 2-O-methylhonokiol suppresses 3T3-L1 adipogenesis by reducing adipogenic and lipogenic execution, accompanied by AMPK-associated metabolic remodeling, kinase signaling changes, selective ER stress-related responses, and restriction of early cell cycle progression. Further pathway-specific studies are required to define the causal contribution of each signaling axis.

## 1. Introduction

Obesity drives a cluster of metabolic disorders that includes type 2 diabetes, non-alcoholic fatty liver disease, and cardiovascular dysfunction [1,2,3]. Much of this risk originates within adipose tissue itself, which expands through hypertrophy of mature adipocytes and through hyperplasia driven by the differentiation of resident progenitor cells. The hyperplastic component reflects adipogenesis, a sequential program in which committed preadipocytes exit the cell cycle, re-enter briefly under hormonal stimulation, and finally mature into lipid-laden adipocytes [4,5,6,7]. Adipose tissue normally provides energy buffering and endocrine signaling. Once adipogenic expansion outpaces metabolic capacity, lipid spillover, ectopic fat deposition, and systemic insulin resistance follow [1,5,8,9]. Agents that restrain adipocyte differentiation without compromising basal cell viability remain useful starting points for metabolic intervention, particularly when they act selectively on the differentiation program rather than through generalized antiproliferative effects.

The differentiation program runs through a defined transcriptional cascade. Adipogenic stimulation produces a rapid, transient induction of C/EBPβ, which binds and activates the promoters of PPARγ and C/EBPα [10,11,12]. PPARγ then partners with C/EBPα to consolidate adipocyte identity and drives expression of lipid-handling and lipogenic proteins, including FABP4 and fatty acid synthase (FASN), which support triglyceride accumulation in lipid droplets [13,14,15]. Pharmacological PPARγ ligands such as the thiazolidinediones illustrate the cost of activating this axis directly. Insulin sensitivity improves under thiazolidinedione treatment, yet adipogenic commitment is reinforced, with weight gain and fluid retention as common adverse effects [16,17,18]. Strategies that suppress the C/EBPβ–PPARγ–C/EBPα cascade, rather than activate PPARγ pharmacologically, present a different starting point for limiting fat storage [19,20,21,22].

Adipogenic commitment is also gated by metabolic and stress checkpoints. AMP-activated protein kinase (AMPK) senses cellular energy status and, when phosphorylated at Thr172, restrains anabolic output through inhibition of mTORC1 and acetyl-CoA carboxylase (ACC) [23,24,25,26]. Because terminal differentiation depends on sustained protein and lipid synthesis, AMPK activation is broadly anti-adipogenic [27,28,29,30]. However, AMPK activation during adipogenesis may not always produce uniform suppression of mTOR-associated readouts, particularly when stress-kinase or ER stress pathways are also engaged. Closely linked to this metabolic axis is mitotic clonal expansion (MCE), the brief proliferative burst in which growth-arrested preadipocytes resume cell cycle progression shortly after MDI induction. Disruption of this early cell cycle remodeling blunts subsequent C/EBPα and PPARγ expression and prevents terminal differentiation [31,32,33]. A third regulatory layer originates at the endoplasmic reticulum. Adipogenesis imposes substantial protein-folding demand, and the unfolded protein response (UPR), particularly the IRE1α–XBP1s and PERK–eIF2α branches, contributes to adipocyte fate decisions and lipid metabolism [34,35,36]. MAPK and AKT signaling are also engaged during differentiation, with ERK, JNK, and AKT influencing the balance between adipogenic progression, stress signaling, and cell survival [22]. Effective inhibition of adipogenesis can therefore engage transcriptional, metabolic, cell cycle, and ER stress components in parallel [37,38].

Natural products and their analogs continue to provide chemical scaffolds for metabolic modulation. Honokiol, a biphenolic neolignan isolated from Magnolia bark, has been reported to regulate inflammation, oxidative stress, mitochondrial function, AMPK signaling, PPARγ activity, and lipid accumulation in adipocyte-related models [39,40]. In the context of adipogenesis and obesity-associated metabolism, honokiol has been suggested to suppress adipocyte differentiation by affecting lipid accumulation, adipogenic transcriptional regulators, and metabolic stress pathways. However, the parent compound also has limitations that may restrict its mechanistic interpretation and translational development, including broad pleiotropic activity, limited target specificity, and biological effects that may vary according to cell type, dose, and treatment window [41,42]. These features make it difficult to determine whether its anti-adipogenic activity is driven by a defined signaling preference or by broader stress-related effects.

The structural distinction between honokiol and 2-O-methylhonokiol may be relevant to their biological behavior. Honokiol contains two phenolic hydroxyl groups that can participate in hydrogen bonding, redox-related interactions, and metabolic conjugation. Methylation of one phenolic hydroxyl group may reduce polarity and alter hydrogen-bonding capacity, potentially affecting membrane permeability, intracellular accumulation, metabolic stability, and target-binding preference. These changes could contribute to a signaling profile that differs from that of the parent compound. In the present study, 2-O-methylhonokiol suppressed lipid accumulation and adipogenic marker expression while altering AMPK, AKT/MAPK, and ER stress-related markers. However, because honokiol and 2-O-methylhonokiol were not directly compared in parallel, the present data cannot determine whether O-methylation enhances, weakens, or redirects anti-adipogenic activity. This structure–activity interpretation should therefore be considered a mechanistic hypothesis requiring direct comparative validation.

## 2. Results

### 2.1. 2-O-Methylhonokiol Suppresses Adipocyte Differentiation and Lipid Accumulation in 3T3-L1 Cells

**Figure 1:**
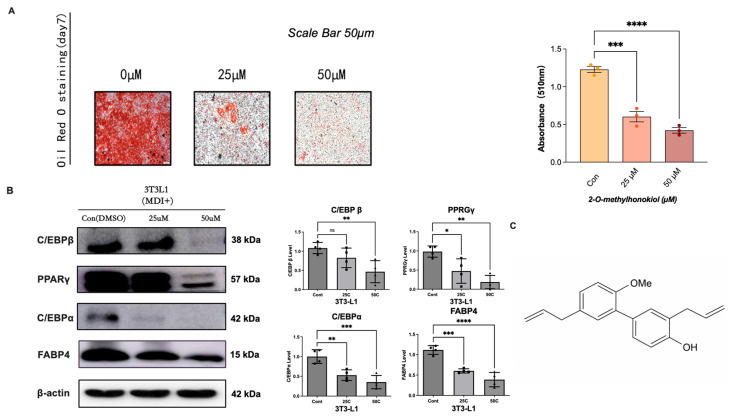
2-O-methylhonokiol inhibits adipocyte differentiation and lipid accumulation in 3T3-L1 cells. 3T3-L1 preadipocytes were induced to differentiate with MDI medium and treated with 2-O-methylhonokiol at 0, 25, or 50 µM during the differentiation period. (**A**) Lipid accumulation was evaluated by Oil Red O staining on day 7 and quantified by elution of bound dye, followed by absorbance measurement at 510 nm. Scale bar = 50 µm. (**B**) Whole-cell lysates were analyzed by immunoblotting for C/EBPβ, PPARγ, C/EBPα, and FABP4. β-Actin was used as a loading control. Densitometric values were normalized to β-actin and expressed relative to the vehicle control. (**C**) Chemical structure of 2-O-methylhonokiol. The vehicle control, Con, consisted of MDI medium supplemented with 0.1% DMSO. The data are presented as mean ± SD from three independent experiments (n = 3). Statistical significance versus the vehicle control was determined by one-way ANOVA followed by Dunnett’s multiple comparisons test; ns, not significant; * *p* < 0.05, ** *p* < 0.01, *** *p* < 0.001, **** *p* < 0.0001.

To determine whether 2-O-methylhonokiol affects adipocyte differentiation, 3T3-L1 preadipocytes were induced with MDI medium and treated with vehicle or 2-O-methylhonokiol during the differentiation period. On day 7, vehicle-treated cells showed strong Oil Red O staining, indicating robust intracellular lipid accumulation. In contrast, cells exposed to 2-O-methylhonokiol displayed visibly reduced lipid deposition in a concentration-dependent manner (Figure 1A). Quantification of extracted Oil Red O dye at 510 nm supported the microscopic observations. Compared with the vehicle control, lipid accumulation was significantly decreased by treatment with 25 µM and 50 µM 2-O-methylhonokiol, with the stronger inhibitory effect observed at 50 µM.

We next examined whether the reduction in lipid accumulation was associated with altered expression of adipogenic regulatory proteins. Immunoblot analysis showed that 50 µM 2-O-methylhonokiol significantly decreased the expression of C/EBPβ, an early transcription factor involved in adipogenic commitment, in differentiating 3T3-L1 cells (Figure 1B). The expression levels of PPARγ and C/EBPα, two major regulators required for terminal adipocyte differentiation, were also reduced following 2-O-methylhonokiol treatment. In addition, FABP4, a marker of mature adipocytes and lipid-handling capacity, was markedly downregulated in treated cells. Densitometric analysis confirmed concentration-dependent decreases in these adipogenic proteins, while β-actin expression remained relatively unchanged across treatment groups. These findings indicate that 2-O-methylhonokiol inhibits 3T3-L1 adipocyte differentiation by reducing lipid accumulation and suppressing key components of the adipogenic transcriptional program.

### 2.2. 2-O-Methylhonokiol Maintains Cell Viability at Lower Anti-Adipogenic Doses

To determine whether the anti-adipogenic activity of 2-O-methylhonokiol is simply a secondary effect of nonspecific cytotoxicity, we evaluated cell viability in 3T3-L1 preadipocytes using a CCK-8 assay. Confluent cells were exposed to a vehicle (0.1% DMSO) or varying concentrations of 2-O-methylhonokiol (25, 50, 75, or 100 µM) for 24 h. This was followed by a 1 h incubation with the CCK-8 reagent and an absorbance measurement at 450 nm.

**Figure 2:**
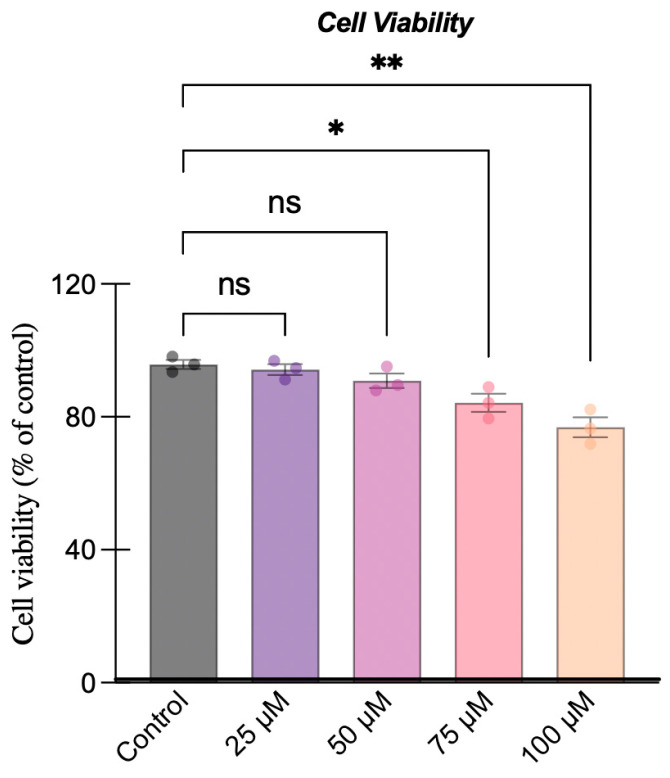
Effect of 2-O-methylhonokiol on 3T3-L1 preadipocyte viability. 3T3-L1 preadipocytes were treated with a vehicle or 2-O-methylhonokiol (25, 50, 75, or 100 µM) for 24 h. Cell viability was assessed via a CCK-8 assay following a 1 h incubation with the reagent and absorbance reading at 450 nm. The data are expressed as a percentage of the vehicle control after background subtraction using reagent-only blank wells. The vehicle control consisted of a growth medium supplemented with 0.1% DMSO. Bars represent the mean ± SD from three independent experiments (n = 3). Statistical significance was determined by a one-way ANOVA followed by Dunnett’s multiple comparisons test against the vehicle control (ns, not significant; * *p* < 0.05, ** *p* < 0.01).

As shown in Figure 2, treating the cells with 25 µM or 50 µM of the compound did not significantly alter viability compared with the vehicle control. However, higher concentrations did lead to a noticeable decline in cell survival, with significant reductions observed at both 75 µM and 100 µM. Together, these results indicate that as long as the concentration is kept at or below 50 µM, the inhibitory effects of 2-O-methylhonokiol on adipogenesis are genuine and not merely attributable to a reduction in basal cell viability.

### 2.3. 2-O-Methylhonokiol Differentially Regulates AKT and MAPK Signaling During 3T3-L1 Adipogenesis

**Figure 3:**
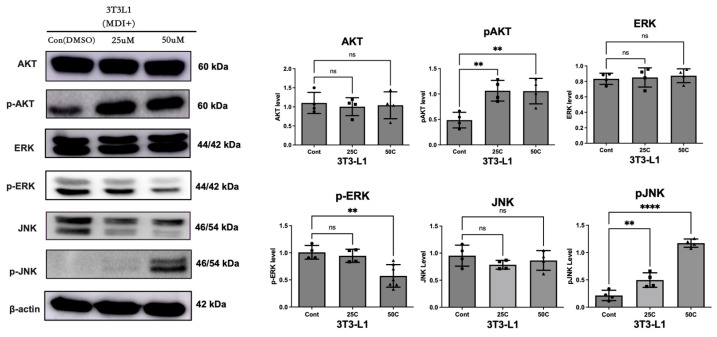
2-O-methylhonokiol differentially regulates AKT and MAPK signaling during 3T3-L1 adipocyte differentiation. 3T3-L1 preadipocytes were induced to differentiate with MDI medium and treated with vehicle control or 2-O-methylhonokiol at 25 µM or 50 µM during the differentiation period. Representative immunoblots are shown for total AKT, phosphorylated AKT, total ERK1/2, phosphorylated ERK1/2, total JNK, phosphorylated JNK, and β-actin. β-Actin was used as the loading control. Densitometric analysis was performed for total protein levels normalized to β-actin and for phosphorylated proteins normalized to their corresponding total protein levels. Values were expressed relative to the vehicle control. The vehicle control consisted of MDI medium supplemented with 0.1% DMSO. The data are presented as mean ± SD from three independent experiments (n = 3). Statistical significance was determined by one-way ANOVA followed by Dunnett’s multiple comparisons test versus the vehicle control; ns, not significant; ** *p* < 0.01, **** *p* < 0.0001.

To further examine the signaling events associated with the anti-adipogenic effect of 2-O-methylhonokiol, differentiating 3T3-L1 cells were treated with 25 µM or 50 µM 2-O-methylhonokiol and analyzed for AKT- and MAPK-associated phosphorylation changes by immunoblotting (Figure 3). Total AKT, ERK1/2, and JNK protein levels were not significantly changed across the treatment groups, indicating that 2-O-methylhonokiol did not substantially alter the overall abundance of these kinases. In contrast, phosphorylation of these proteins was differentially regulated. AKT phosphorylation at Ser473 was significantly increased at both 25 µM and 50 µM, whereas total AKT expression remained largely unchanged. Because downstream adipogenic and lipogenic outputs were suppressed under the same conditions, this increase in AKT phosphorylation should be interpreted as a phosphorylation event associated with 2-O-methylhonokiol treatment rather than as definitive evidence of canonical pro-adipogenic AKT pathway activation. ERK1/2 phosphorylation showed the opposite pattern, with a reduction after treatment and a statistically significant decrease at 50 µM, indicating that higher concentrations of 2-O-methylhonokiol attenuate ERK1/2 activation. JNK phosphorylation was markedly elevated in response to 2-O-methylhonokiol, with a stronger increase at 50 µM than at 25 µM, while total JNK expression remained stable. Together, these data indicate that 2-O-methylhonokiol selectively modifies kinase phosphorylation rather than broadly reducing kinase expression, producing a signaling profile characterized by increased AKT and JNK phosphorylation together with reduced detectable ERK1/2 phosphorylation. This altered balance of AKT/MAPK signaling may contribute to the suppression of adipogenic differentiation observed in 2-O-methylhonokiol-treated 3T3-L1 cells.

### 2.4. 2-O-Methylhonokiol Is Associated with Increased IRE1α Protein Abundance and XBP1s Accumulation During 3T3-L1 Differentiation

**Figure 4:**
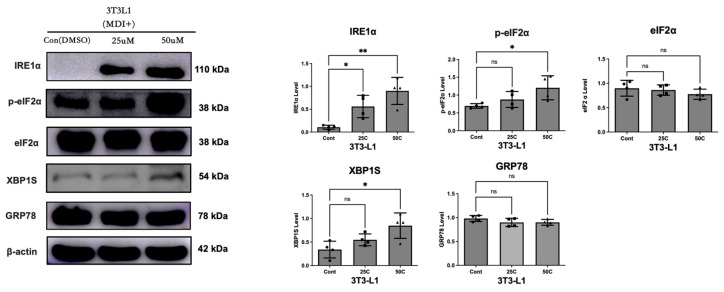
2-O-methylhonokiol is associated with increased IRE1α protein abundance and XBP1s accumulation in differentiating 3T3-L1 cells. 3T3-L1 preadipocytes were induced to differentiate with MDI medium and treated with vehicle control or 2-O-methylhonokiol at 25 μM or 50 μM during the differentiation period. Representative immunoblots are shown for IRE1α, phosphorylated eIF2α, total eIF2α, XBP1s, GRP78, and β-actin. β-Actin was used as the loading control. Densitometric quantification was performed as IRE1α/β-actin, p-eIF2α/eIF2α, total eIF2α/β-actin, XBP1s/β-actin, and GRP78/β-actin, with values normalized to the vehicle control. The vehicle control consisted of MDI medium supplemented with 0.1% DMSO. The data are presented as mean ± SD from three independent experiments (n = 3). Statistical significance was determined by one-way ANOVA followed by Dunnett’s multiple comparisons test versus the vehicle control; ns, not significant; * *p* < 0.05, ** *p* < 0.01.

In order to ascertain the impact of 2-O-methylhonokiol on ER stress-associated signalling during adipocyte differentiation, differentiating 3T3-L1 cells were treated with 25 μM or 50 μM 2-O-methylhonokiol and analysed for proteins related to the IRE1α–XBP1s axis and eIF2α signalling (Figure 4). The present study demonstrated that the abundance of the IRE1α protein was significantly increased by 2-O-methylhonokiol at both concentrations, with a more pronounced elevation at 50 μM. In line with this response, XBP1s expression was also increased, reaching statistical significance at 50 μM, whereas the 25 μM treatment produced only a modest, non-significant change. Phosphorylation of eIF2α demonstrated a modest increase and attained statistical significance only at 50 μM, while total eIF2α protein levels remained unaltered across all experimental groups. Conversely, GRP78 demonstrated no augmentation following 2-O-methylhonokiol treatment, exhibiting no statistically significant alteration.

These results indicate that 2-O-methylhonokiol does not elicit a uniform ER stress response during 3T3-L1 differentiation. Instead, the response is characterised primarily by increased IRE1α abundance and XBP1s induction, accompanied by a modest increase in eIF2α phosphorylation at the higher concentration, but without detectable GRP78 upregulation. This selective ER stress-related profile, defined by increased total IRE1α protein abundance, XBP1s accumulation at 50 µM, and increased eIF2α phosphorylation at 50 µM, may be associated with impaired adipogenic progression in 2-O-methylhonokiol-treated cells.

### 2.5. 2-O-Methylhonokiol Modulates Cell Cycle Distribution During Early Adipogenic Differentiation

**Figure 5:**
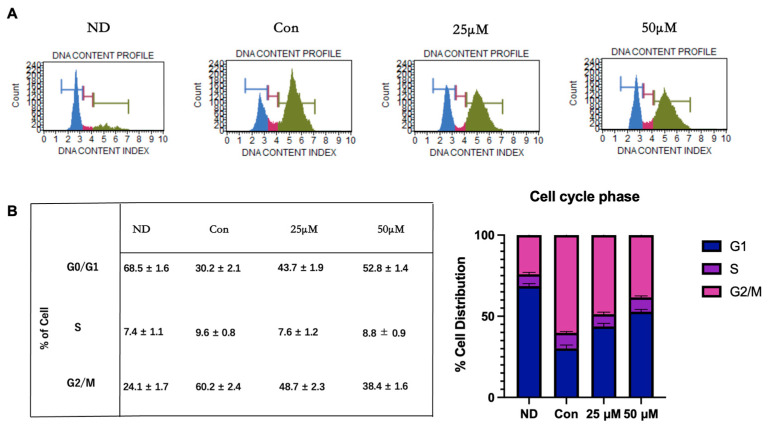
2-O-methylhonokiol attenuates MDI-induced cell cycle redistribution in 3T3-L1 preadipocytes. Confluent 3T3-L1 preadipocytes were maintained in growth medium without MDI induction or stimulated with MDI differentiation medium in the presence or absence of 2-O-methylhonokiol at 25 or 50 µM for 24 h. Cells were fixed in 70% ethanol, stained with Muse™ Cell Cycle Reagent, and analyzed using a Muse™ Cell Analyzer. (**A**) Representative DNA-content histograms showing cell cycle distribution across G0/G1, S, and G2/M phases. (**B**) Quantitative analysis of cell cycle phase distribution. MDI induction promoted a shift from G0/G1 toward G2/M, whereas 2-O-methylhonokiol partially restored the G0/G1 population and reduced the G2/M fraction. The data are presented as mean ± SD from three independent experiments (n = 3). Statistical significance was determined by one-way ANOVA followed by Tukey’s multiple-comparisons test. ND, non-differentiated cells; Con, MDI-induced vehicle control.

Mitotic clonal expansion is an early proliferative event required for adipogenic differentiation, during which confluent 3T3-L1 preadipocytes re-enter the cell cycle after MDI stimulation. To determine whether 2-O-methylhonokiol affects this early cell cycle response, DNA content profiles were analyzed 24 h after MDI induction (Figure 5). Non-differentiated cells were mainly retained in G0/G1 phase, consistent with a confluent growth-arrested state. MDI induction markedly shifted the cell cycle profile, decreasing the G0/G1 population from 68.5 ± 1.6% to 30.2 ± 2.1% and increasing the G2/M fraction from 24.1 ± 1.7% to 60.2 ± 2.4%. Treatment with 2-O-methylhonokiol partially reversed this redistribution in a concentration-dependent manner, increasing the G0/G1 population to 43.7 ± 1.9% and 52.8 ± 1.4% at 25 and 50 µM, respectively, while reducing the G2/M fraction to 48.7 ± 2.3% and 38.4 ± 1.6%. The S-phase fraction showed only modest changes among the MDI-induced groups, with values of 9.6 ± 0.8%, 7.6 ± 1.2%, and 8.8 ± 0.9% in the Con, 25 µM, and 50 µM groups, respectively. These findings indicate that 2-O-methylhonokiol limits MDI-induced cell cycle remodeling during early adipogenic differentiation, maintaining a larger proportion of cells in G0/G1 and reducing progression toward a G2/M-enriched state associated with mitotic clonal expansion.

### 2.6. 2-O-Methylhonokiol Increases AMPKα Phosphorylation and Reduces FASN Expression During Adipocyte Differentiation

**Figure 6:**
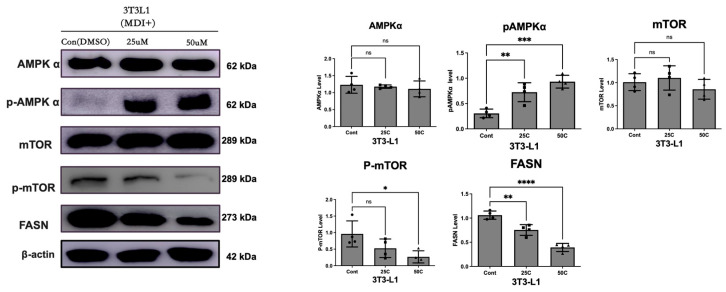
2-O-methylhonokiol activates AMPK signaling and suppresses FASN expression in differentiating 3T3-L1 cells. 3T3-L1 preadipocytes were induced to differentiate with MDI medium and treated with vehicle control or 2-O-methylhonokiol at 25 µM or 50 µM during differentiation. Representative immunoblots are shown for AMPKα, phosphorylated AMPKα, mTOR, phosphorylated mTOR, FASN, and β-actin. β-Actin was used as the loading control for total protein analysis. Densitometric quantification was performed by normalizing total protein levels to β-actin and phosphorylated proteins to their corresponding total protein levels. Values are expressed relative to the vehicle-treated control and presented as mean ± SD from three independent experiments (n = 3). Statistical significance was assessed by one-way ANOVA followed by Dunnett’s multiple comparisons test versus the vehicle control; ns, not significant; * *p* < 0.05, ** *p* < 0.01, *** *p* < 0.001, **** *p* < 0.0001.

The effect of 2-O-methylhonokiol on AMPK- and mTOR-associated metabolic signaling was examined in differentiating 3T3-L1 cells treated with 25 µM or 50 µM of the compound (Figure 6). Total AMPKα protein levels remained largely unchanged across treatment groups, whereas AMPKα phosphorylation increased markedly after treatment. This pattern indicates that 2-O-methylhonokiol increased AMPKα phosphorylation without altering total AMPKα abundance. Similarly, total mTOR protein expression was not significantly affected by the treatment; however, phosphorylated mTOR (p-mTOR) levels were significantly decreased following treatment with 50 µM 2-O-methylhonokiol. Thus, while total mTOR abundance remained stable, AMPK activation was accompanied by a clear concentration-dependent suppression of mTOR phosphorylation, culminating in a significant reduction at the highest anti-adipogenic dose. FASN expression was likewise significantly reduced in cells treated with 2-O-methylhonokiol, with the strongest suppression observed at 50 µM. Because FASN is a major lipogenic enzyme required for fatty acid synthesis during adipocyte maturation, its reduction is consistent with impaired lipid accumulation and attenuated adipogenic progression. Taken together, these results show that 2-O-methylhonokiol increases AMPKα phosphorylation and suppresses both p-mTOR and FASN expression in differentiating 3T3-L1 cells.

## 3. Discussion

**Figure 7:**
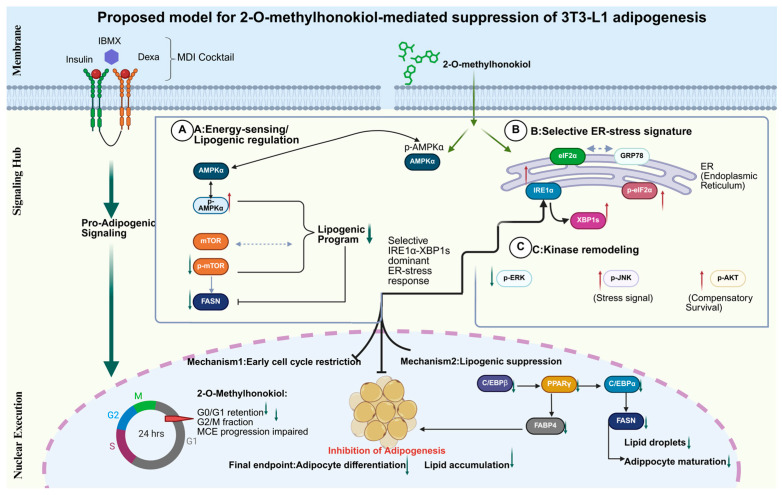
Proposed model for 2-O-methylhonokiol-associated suppression of 3T3-L1 adipogenesis. MDI stimulation promotes pro-adipogenic signaling and drives adipocyte differentiation. 2-O-methylhonokiol is proposed to suppress this process through interconnected signaling changes: (**A**) energy-sensing/lipogenic regulation, characterized by increased p-AMPKα and reduced FASN, with reduced p-mTOR at 50 µM; (**B**) a selective ER stress-related signature involving increased IRE1α protein abundance, XBP1s accumulation, and increased p-eIF2α; and (**C**) kinase remodeling, including decreased p-ERK and increased p-JNK, with increased p-AKT potentially reflecting an adaptive survival-associated phosphorylation event. These changes are associated with early cell cycle restriction and suppression of the C/EBPβ–PPARγ–C/EBPα adipogenic axis, resulting in reduced FABP4/FASN expression, lipid droplet formation, and adipocyte maturation.

2-O-methylhonokiol, a 2-O-methylated derivative of honokiol, markedly suppressed adipocyte differentiation and lipid accumulation in 3T3-L1 cells at concentrations that did not compromise basal cell viability. This distinction is important because the observed anti-adipogenic effect is unlikely to reflect nonspecific cytotoxicity. Instead, the updated findings support a model in which 2-O-methylhonokiol interferes with the signaling pathways required for adipogenic commitment. MDI-induced differentiation normally depends on early mitogenic signaling, mitotic clonal expansion, activation of the C/EBPβ–PPARγ–C/EBPα transcriptional cascade, and subsequent induction of lipogenic and adipocyte maturation markers. 2-O-methylhonokiol disrupted several of these processes simultaneously. At the upstream level, it increased AMPKα phosphorylation, altered ER stress-related signaling, reduced ERK phosphorylation, increased JNK phosphorylation, and elevated AKT phosphorylation [4,43,44]. These changes were accompanied by impaired cell cycle progression and reduced expression of C/EBPβ, PPARγ, C/EBPα, FABP4, and FASN. Thus, the most consistent interpretation is that 2-O-methylhonokiol does not block adipogenesis through a single linear pathway, but rather induces a stress-adaptive and anti-lipogenic state that prevents the normal transition from preadipocyte to mature lipid-storing adipocyte. This integrated mechanism, linking upstream signaling remodeling to downstream cell cycle and transcriptional restriction, is summarized schematically in Figure 7. While the parent compound honokiol has been documented to attenuate adipogenesis via direct PPARγ modulation or canonical AMPK pathways, the functional consequences of its structurally modified analog, 2-O-methylhonokiol, reveal a highly distinct and sophisticated mechanical signature. The ER stress-related profile should also be interpreted cautiously. 2-O-methylhonokiol increased total IRE1α protein abundance and XBP1s accumulation, together with increased eIF2α phosphorylation at 50 µM, whereas GRP78 was not increased. This pattern suggests a selective ER stress-related response rather than a uniform canonical UPR. However, total IRE1α protein abundance does not directly demonstrate activation of the IRE1α kinase or endoribonuclease activity. Because phosphorylated IRE1α and Xbp1 mRNA splicing were not examined, the present data cannot establish direct activation of the IRE1α–XBP1 branch. Therefore, the findings should be interpreted as increased IRE1α protein abundance and XBP1s accumulation associated with the anti-adipogenic response, rather than definitive evidence that IRE1α activation functionally mediates the phenotype [45,46]. Furthermore, this derivative uncouples mitogenic hormonal inputs from structural execution by co-activating a compensatory p-AKT survival loop while executing stringent G0/G1 cell cycle restriction during the earliest windows of mitotic clonal expansion. This multifaceted, non-canonical stress reconfiguration underscores the uniqueness of the 2-O-methylhonokiol scaffold as a specialized metabolic modulator.

AMPK activation represents a major component of this response. As a metabolic checkpoint, AMPK limits anabolic processes under conditions of energetic or cellular stress, and its activation is generally unfavorable for adipocyte differentiation and lipid biosynthesis. The increase in p-AMPKα observed following 2-O-methylhonokiol treatment therefore provides a plausible upstream explanation for the reduction in lipid accumulation [27]. This interpretation is further supported by the decrease in FASN, a key enzyme involved in de novo fatty acid synthesis. However, the present data should not be reduced to a simple AMPK–mTORC1 axis. Although the updated model includes reduced p-mTOR, 2-O-methylhonokiol also induced ER stress- and stress kinase-related changes, indicating that AMPK activation occurs within a broader signaling reconfiguration [44]. In this context, the ER stress-related profile is particularly relevant. 2-O-methylhonokiol increased total IRE1α protein abundance and XBP1s accumulation and also elevated p-eIF2α at 50 µM, whereas GRP78 did not show a comparable increase. This pattern suggests a selective ER stress-related response rather than a uniform canonical UPR. However, total IRE1α protein abundance does not directly demonstrate activation of the IRE1α kinase or endoribonuclease activity, and XBP1s protein accumulation alone does not fully define the extent of Xbp1 mRNA splicing. Because phosphorylated IRE1α, ER stress-positive controls, and RT-PCR-based Xbp1s/Xbp1u analysis were not included in the present study, this response should be interpreted cautiously. Thus, the current evidence supports an association between increased IRE1α protein abundance, XBP1s accumulation, p-eIF2α elevation, and impaired adipogenic progression, but does not establish that the IRE1α–XBP1 branch functionally mediates the anti-adipogenic phenotype. Future studies using p-IRE1α detection, tunicamycin or thapsigargin controls, and Xbp1 splicing assays will be required to validate this pathway more directly. In our model, the lack of GRP78 induction deprives the cells of an adaptive chaperoning buffer, effectively locking the preadipocytes into a restrictive, chronic stress state. Concurrently, this persistent IRE1α activation feeds into the p-JNK stress cascade, contributing to a restrictive stress-associated state that halts mitotic clonal expansion and prevents adipogenic execution while maintaining basal cell viability via a compensatory p-AKT loop. The role of IRE1α–XBP1s signaling in adipogenesis is context-dependent: adaptive activation may support adipocyte maturation by improving ER folding capacity and lipid-handling function, whereas sustained or dysregulated ER stress can restrict differentiation [47]. In the present study, IRE1α–XBP1s activation occurred alongside increased p-JNK, suppression of adipogenic transcription factors, and reduced lipid accumulation, making a restrictive stress state more likely than a differentiation-supportive adaptive response. Nevertheless, the current evidence remains associative; whether IRE1α–XBP1s activation directly contributes to the anti-adipogenic phenotype or arises secondarily from metabolic perturbation requires targeted inhibition or loss-of-function experiments [48].

The kinase and cell cycle data provide a mechanistic link between upstream signaling changes and downstream transcriptional suppression. Reduced ERK phosphorylation is consistent with weakened mitogenic input during the early phase of adipogenesis, when proliferative signaling is required for mitotic clonal expansion. Increased JNK phosphorylation further indicates activation of a stress-responsive program that may reinforce the differentiation block. The increase in AKT phosphorylation presents an apparent divergence from the expected pro-adipogenic role of canonical insulin–PI3K–AKT signaling. In this study, however, increased AKT Ser473 phosphorylation occurred together with reduced lipid accumulation and decreased expression of PPARγ, C/EBPα, FABP4, and FASN. This pattern suggests that AKT phosphorylation was not translated into a productive adipogenic output under 2-O-methylhonokiol treatment. One possible interpretation is that increased p-AKT reflects an adaptive survival-associated phosphorylation response in differentiating cells exposed to metabolic or stress-related perturbation. However, because downstream AKT substrates such as PRAS40, GSK-3β, FOXO1, TSC2, and S6K1 were not directly assessed, the present data cannot determine whether AKT phosphorylation reflects survival signaling, metabolic feedback, or pathway-specific uncoupling from adipogenesis [49,50]. The preservation of basal cell viability suggests that 2-O-methylhonokiol does not suppress adipogenesis through nonspecific cytotoxicity. Instead, the elevated p-AKT signal may represent a stress-adaptive survival response associated with IRE1α–XBP1s activation and increased JNK phosphorylation. However, this AKT activation appears to be uncoupled from adipogenic execution. By disrupting mitotic clonal expansion and suppressing the PPARγ/C/EBPα transcriptional axis, 2-O-methylhonokiol may block the conversion of upstream AKT signaling into terminal adipocyte differentiation and lipogenic programming. In this setting, p-AKT is not absent, but its biological output is redirected from adipogenesis toward cellular adaptation [38].

At the cellular level, 2-O-methylhonokiol increased G0/G1 retention and reduced progression into the S and G2/M phases, indicating impaired early cell cycle progression. Because mitotic clonal expansion is required for efficient 3T3-L1 adipogenesis, this defect would be expected to weaken the induction of C/EBPβ and subsequently reduce PPARγ and C/EBPα expression [38]. The observed decrease in FABP4 and FAS then reflects a failure to establish the mature adipocyte program, explaining the reduction in lipid droplet formation and lipid accumulation. Although this integrated model is consistent with the current dataset, a causal hierarchy cannot yet be established. Future studies utilizing AMPK inhibition, IRE1α pathway modulation, Xbp1 splicing analysis, JNK or ERK modulation, and restoration of PPARγ activity would be necessary to determine which signaling nodes are required for the anti-adipogenic effect of 2-O-methylhonokiol. In addition, downstream AKT substrates such as PRAS40, GSK-3β, FOXO1, TSC2, and S6K1 were not directly examined in this study. Future work assessing these substrates, together with AKT inhibition or genetic modulation, will be required to determine whether increased AKT phosphorylation reflects survival signaling, metabolic feedback, or pathway-specific uncoupling from adipogenic execution [38,51]. Validation in primary preadipocytes and in vivo adipose models would also be needed to define the physiological relevance of this honokiol-derived scaffold [4]. Because the direct molecular targets of 2-O-methylhonokiol remain unknown, future studies will further incorporate molecular docking and structure-activity relationship modeling to prioritize plausible interactions with functionally relevant candidate proteins identified through these pathway-directed experiments. The resulting predictions will require confirmation through biochemical binding assays and targeted functional validation to distinguish direct target engagement from secondary signaling responses.

## 4. Materials and Methods

### 4.1. Culture of Cells and Differentiation

Murine 3T3-L1 preadipocytes were obtained from the American Type Culture Collection (ATCC) (Manassas, VA, USA). 3T3-L1 preadipocytes were cultured in Dulbecco’s modified Eagle medium (DMEM) supplemented with 10% calf serum and 1% penicillin–streptomycin at 37 °C in a humidified atmosphere of 5% CO_2_ until confluent. Post-confluent cultures were maintained in the same medium for an additional 48 h to allow growth arrest. Differentiation was then induced with MDI medium containing 0.5 mM 3-isobutyl-1-methylxanthine (IBMX), 0.25 µM dexamethasone, and 10 µg/mL insulin for 48 h. The induction medium was replaced with DMEM containing 10% fetal bovine serum, 1% penicillin–streptomycin, and 10 µg/mL insulin, and cells were maintained for a further 48 h. From day 4 to day 7, the maintenance medium was renewed every day. On day 7, lipid accumulation and adipocyte morphology were examined by bright-field microscopy at 40× magnification. In all differentiation experiments, 2-O-methylhonokiol (25 or 50 µM) or vehicle (0.1% DMSO, final concentration) was added throughout the differentiation period unless otherwise stated.

### 4.2. Cell Viability Assay

Cell viability was assessed with a Cell Counting Kit-8 (CCK-8; D-Plus™ CCK cell viability assay kit, DonginLS, Seoul, Republic of Korea; Cat. No. CCK-3000) according to the manufacturer’s instructions. 3T3-L1 preadipocytes were seeded in 96-well plates at 5 × 10^3^ cells/well in 100 µL of complete growth medium and allowed to attach overnight. Cells were treated with vehicle (0.1% DMSO) or 2-O-methylhonokiol (25, 50, 75, or 100 µM) for 24 h, with the final DMSO concentration held constant across all groups. CCK-8 reagent (10 µL) was then added to each well and plates were incubated at 37 °C for 1 h in the dark. Absorbance was measured at 450 nm on a microplate reader (Synergy HTX Multi-Mode Microplate Reader, BioTek Instruments, Winooski, VT, USA). Wells containing medium and CCK-8 reagent without cells were used as blanks for background subtraction. Cell viability was expressed as a percentage of the vehicle control and calculated as follows:

$$Viability\left(\%\right)=\frac{A_{treated}-A_{blank}}{A_{control}-A_{blank}}\times 100$$

All experiments were performed with three independent biological replicates (n = 3) and three technical replicates per condition within each experiment.

### 4.3. Western Blot Analysis

Whole-cell lysates were prepared in ice-cold RIPA buffer (50 mM Tris-HCl, pH 7.6; 150 mM NaCl; 1% Triton X-100; 1% sodium deoxycholate) supplemented with protease and phosphatase inhibitors. Protein concentration was determined using the Bradford assay, and equal amounts of protein were resolved on 6–15% SDS–PAGE gels and transferred to BioTrace NT nitrocellulose membranes (Pall Life Sciences, Port Washington, MA, USA). Membranes were blocked for 1 h at room temperature in 5% BSA/TBST for phospho-specific antibodies or in 5% skim milk/TBST for non-phosphorylated targets. Primary antibodies were incubated overnight at 4 °C at a dilution of 1:1000, followed by three washes with TBST for 5 min each and incubation with HRP-conjugated secondary antibodies at 1:10,000 for 1 h at room temperature. Signals were developed using Clarity Western ECL Substrate (Bio-Rad Laboratories, Hercules, CA, USA) and captured under non-saturating conditions. Band intensities were quantified using ImageJ v1.54i. Total protein levels were normalized to β-actin, whereas phosphorylated proteins were normalized to their corresponding total protein levels.

Primary antibodies were obtained from Santa Cruz Biotechnology (Santa Cruz, CA, USA): C/EBPβ (sc-7962), FABP4 (sc-18661), IRE1α (sc-31199), and GRP78 (sc-1050). The following primary antibodies were obtained from Cell Signaling Technology (Danvers, MA, USA): PPARγ (2443S), C/EBPα (2295S), XBP1s (83418S), AMPKα (2532S), phospho-AMPKα Thr172 (2535S), mTOR (2983S), phospho-mTOR Ser2448 (5536S), FAS/FASN (3180S), AKT (5298M), phospho-AKT Ser473 (4060S), p44/42 MAPK/ERK1/2 (4695S), phospho-p44/42 MAPK/ERK1/2 Thr202/Tyr204 (4370S), SAPK/JNK (9252S), phospho-SAPK/JNK Thr183/Tyr185 (4668S), eIF2α (5324S), phospho-eIF2α Ser51 (3398S), and β-actin (13E5; 4970).

### 4.4. Oil Red O Staining

On day 7 of differentiation, cultures were rinsed three times with phosphate-buffered saline (PBS) and fixed in 3.7% paraformaldehyde for 60 min at room temperature. After fixation, cells were washed three times with PBS and stained for 1 h with a 0.5% (*w*/*v*) Oil Red O working solution prepared in isopropanol. Excess stain was removed by three washes with deionized water. Stained cells were imaged on an IX73 bright-field inverted microscope (Olympus, Tokyo, Japan) at 100× magnification, with five random fields captured per well. For quantification, bound dye was extracted with 500 µL isopropanol for 10 min at room temperature and absorbance was measured at 510 nm on a SpectraMax microplate reader (Molecular Devices, San Jose, CA, USA).

### 4.5. Cell Cycle Analysis by Flow Cytometry

3T3-L1 preadipocytes were seeded at 1 × 10^5^ cells per 60 mm dish and grown to confluence. Cells were assigned to four groups: a non-differentiated (ND) group maintained in growth medium without MDI stimulation, and three MDI-induced groups treated with vehicle (0.1% DMSO), 25 µM 2-O-methylhonokiol, or 50 µM 2-O-methylhonokiol. After 24 h, cells were harvested, washed with PBS, and fixed in cold 70% ethanol. Fixed cells were stained with Muse™ Cell Cycle Reagent (Luminex, Austin, TX, USA) for 30 min in the dark, and DNA content was analyzed on a Muse™ Cell Analyzer (Merck Millipore, Billerica, MA, USA). The percentage of cells in G0/G1, S, and G2/M phases was determined from the resulting histograms.

### 4.6. Statistical Analyses

The data are expressed as mean ± standard deviation (SD) from at least three independent experiments unless stated otherwise. All analyses were performed in GraphPad Prism 10 (GraphPad Software, Boston, MA, USA). Most experiments used three groups: vehicle-treated Control (MDI + 0.1% DMSO), 25 µM 2-O-methylhonokiol, and 50 µM 2-O-methylhonokiol. Two experiments had a different design: the CCK-8 viability assay additionally included 75 µM and 100 µM 2-O-methylhonokiol, and the cell cycle analysis included a non-differentiated (ND) group (confluent 3T3-L1 cells in growth medium without MDI induction) as an unstimulated baseline alongside the Control, 25 µM, and 50 µM groups. Group comparisons were performed by one-way analysis of variance (ANOVA) followed by Dunnett’s test against the vehicle Control or Tukey’s test for all pairwise comparisons. Two-group comparisons used an unpaired two-tailed Student’s *t*-test. *p* < 0.05 was considered statistically significant. Statistical significance is indicated as * *p* < 0.05, ** *p* < 0.01, *** *p* < 0.001, and **** *p* < 0.0001.

## 5. Conclusions

2-O-Methylhonokiol, a 2-O-methylated derivative of honokiol, suppresses MDI-induced adipocyte differentiation in 3T3-L1 preadipocytes. This inhibition is reflected by reduced lipid accumulation and coordinated downregulation of key adipogenic markers, including C/EBPβ, PPARγ, C/EBPα, FABP4, and FASN. At the signaling level, 2-O-methylhonokiol increases AMPKα phosphorylation and decreases FASN expression, while total AMPKα and mTOR remain largely unchanged, accompanied by a significant suppression of mTOR phosphorylation at 50 µM. These findings suggest that 2-O-methylhonokiol effectively reprograms metabolic signaling and lipogenic capacity through the joint inactivation of p-mTOR and FASN. 2-O-methylhonokiol also modifies kinase activity, increasing AKT and JNK phosphorylation while reducing the detectable phosphorylated ERK signal. Because ERK1 and ERK2 were not always simultaneously resolved, this change is best interpreted as altered ERK-associated phosphorylation rather than isoform-specific regulation. 2-O-methylhonokiol further induces a selective ER stress-related response characterized by increased IRE1α expression, XBP1s induction, increased p-eIF2α, unchanged total eIF2α, and no compensatory increase in GRP78. In addition, cell cycle analysis indicates that 2-O-methylhonokiol attenuates the early MDI-induced redistribution associated with mitotic clonal expansion by preserving a larger G0/G1 population and reducing progression toward later cell cycle phases. Together, these results indicate that 2-O-methylhonokiol interferes with adipogenesis through coordinated effects on adipogenic transcription, lipogenic protein expression, AMPK-associated metabolic signaling, ER stress-related and kinase-associated signaling changes, and early cell cycle remodeling. Further studies using pathway-specific inhibition or genetic loss-of-function approaches, followed by validation in primary adipocytes and in vivo models, will be necessary to determine which of these changes are causally required for the anti-adipogenic activity of this honokiol-derived compound.

## Data Availability

The original contributions presented in this study are included in the article. Further inquiries can be directed to the corresponding author.

## Abbreviations

The following abbreviations are used in this manuscript:

3T3-L1	Mouse preadipocyte cell line
AKT	Protein kinase B
AMPK	AMP-activated protein kinase
ANOVA	Analysis of variance
BSA	Bovine serum albumin
CCK-8	Cell Counting Kit-8
C/EBPα	CCAAT/enhancer-binding protein alpha
C/EBPβ	CCAAT/enhancer-binding protein beta
Con	Control
DMEM	Dulbecco’s modified Eagle medium
DMSO	Dimethyl sulfoxide
ECL	Enhanced chemiluminescence
eIF2α	Eukaryotic translation initiation factor 2 alpha
ER	Endoplasmic reticulum
ERK	Extracellular signal-regulated kinase
FABP4	Fatty acid-binding protein 4
FAS/FASN	Fatty acid synthase
G0/G1	Gap 0/gap 1 phase
G2/M	Gap 2/mitotic phase
GRP78	Glucose-regulated protein 78
HRP	Horseradish peroxidase
IBMX	3-isobutyl-1-methylxanthine
IRE1α	Inositol-requiring enzyme 1 alpha
JNK	c-Jun N-terminal kinase
MAPK	Mitogen-activated protein kinase
MCE	Mitotic clonal expansion
MDI	3-isobutyl-1-methylxanthine, dexamethasone, and insulin
mTOR	Mechanistic target of rapamycin
mTORC1	Mechanistic target of rapamycin complex 1
ND	Non-differentiated
ns	Not significant
PBS	Phosphate-buffered saline
PPARγ	Peroxisome proliferator-activated receptor gamma
RIPA	Radioimmunoprecipitation assay
SD	Standard deviation
SDS–PAGE	Sodium dodecyl sulfate–polyacrylamide gel electrophoresis
TBST	Tris-buffered saline with Tween 20
UPR	Unfolded protein response
XBP1s	Spliced X-box binding protein 1
